# Deep Learning for the Detection of Corneal Perforation on Anterior-Segment Optical Coherence Tomography in Microbial Keratitis

**DOI:** 10.3390/bioengineering13060649

**Published:** 2026-05-30

**Authors:** Lucia H. Rhode, Kamini N. Reddy, Folahan Ibukun, Subeesh Kuyyadiyil, Elesh Jain, Gautam S. Parmar, Rama Chellappa, Nakul S. Shekhawat

**Affiliations:** 1 Department of Electrical and Computer Engineering, Whiting School of Engineering, Johns Hopkins University, Baltimore, MD 21218, USA; lrhode1@jh.edu (L.H.R.);; 2 Wilmer Eye Institute, Johns Hopkins University School of Medicine, Baltimore, MD 21287, USA; 3 SNC Chitrakoot, Chitrakoot 485334, Madhya Pradesh, India; 4 Department of Biomedical Engineering, School of Medicine, Johns Hopkins University, Baltimore, MD 21218, USA

**Keywords:** anterior segment optical coherence tomography, microbial keratitis, corneal perforation, deep learning, artificial intelligence

## Abstract

*Purpose*: The purpose of this study was to develop and evaluate deep learning models for automated detection of corneal perforation in microbial keratitis using anterior segment optical coherence tomography (ASOCT) images. *Methods*: We enrolled 150 patients with microbiologically confirmed keratitis. Contralateral healthy eyes served as controls. Ground-truth labels for perforation were established following consensus grading by two masked ophthalmologist graders. A ResNet-34 backbone was used to encode six radial ASOCT scans of an eye independently and mean-pooled into a single eye-level prediction for classification of the presence or absence of corneal perforation. Four model variants were trained. Models differed in the inclusion of healthy controls and stochastic masking of non-corneal anterior segment anatomy during training. All four model variants were evaluated with 5-fold patient-level cross-validation, and the recommended model was chosen on pooled out-of-fold (OOF) test performance. *Results*: All four model variants achieved high discrimination, with pooled OOF test receiver operating characteristic area under the curve (ROC AUC) between 0.924 and 0.971. The best-performing model (Model 3), which did not include healthy controls or stochastic masking of the inferior image portion during training, achieved an ROC AUC of 0.971 (95% CI, 0.943–0.993), average precision (AP) of 0.863 (95% CI, 0.713–0.963), sensitivity of 0.875 (95% CI, 0.727–1.000), specificity of 0.913 (95% CI, 0.858–0.959), and F1 of 0.750 (95% CI, 0.609–0.870) at the validation-derived Youden threshold. The addition of healthy contralateral eyes to the training set did not improve pooled OOF test metrics, and stochastic inferior blackout produced opposing effects in the two training cohort settings. In the infected-only cohort, it reduced both ROC AUC and AP, whereas in the +healthy cohort, it increased ROC AUC and substantially increased AP. *Conclusions*: Deep learning models achieved high diagnostic accuracy for detecting corneal perforation on ASOCT imaging in eyes with microbial keratitis. These findings support the potential role of automated ASOCT analysis as a clinical decision-support tool for identifying this vision-threatening complication.

## 1. Introduction

Microbial keratitis is the leading cause of corneal blindness and the fifth leading cause of blindness globally [1,2]. The disease burden is particularly severe in low- and middle-income countries, where agricultural occupations, limited access to eye care, and delayed presentation contribute to poor visual outcomes [3,4]. Corneal perforation, which is a full-thickness defect in the corneal stroma caused by inflammatory degradation of stromal collagen fibers in response to severe infection, is associated with profound corneal damage and is one of the most devastating complications of microbial keratitis [5]. Left untreated, perforation can lead to prolapse of intraocular contents, endophthalmitis, and loss of the eye [6,7]. Management options for perforation range from observation with medical therapy alone for small perforations with iris plugging to procedural interventions for larger perforations, such as cyanoacrylate gluing [8], Tenon patch graft [9,10], multilayered amniotic membrane grafting [11,12], or tectonic keratoplasty [5,13,14]. Accurate identification of corneal perforation is therefore essential for corneal surgeons to determine the necessity, optimal method, and timing of repair and prevent major adverse outcomes.

Anterior segment optical coherence tomography (ASOCT) has emerged as a clinically useful imaging modality for evaluating corneal infections due to its high-resolution cross-sectional visualization of stromal architecture, infectious infiltrate depth, and more serious anatomic damage that may not be apparent on slit lamp examination alone [15,16,17,18,19,20,21]. A recent diagnostic concordance study demonstrated that ophthalmologists interpreting ASOCT images were able to detect corneal perforation with substantially greater sensitivity than slit lamp examination, with ASOCT identifying perforation in 16% of eyes compared to 8% via slit lamp biomicroscopy [21]. Ophthalmologists interpreting ASOCT images also demonstrated near-perfect inter-grader agreement (κ = 0.98) for detection of perforation, indicating that ASOCT could serve as an objective, reproducible, and scalable assessment tool for microbial keratitis. However, ophthalmologist interpretation of ASOCT images requires expertise that is not uniformly available, particularly in remote or resource-limited settings where microbial keratitis is most prevalent.

Deep learning approaches have demonstrated considerable promise across ophthalmic imaging modalities, with successful applications including diabetic retinopathy screening, glaucoma detection, and age-related macular degeneration assessment [22,23,24]. Computer vision algorithms performing automated assessment of ASOCT images could enable rapid, objective, and clinically actionable identification of corneal perforation in microbial keratitis, potentially bridging the expertise gap across varying practice environments. However, the application of deep learning to anterior segment pathology, particularly in the context of microbial keratitis, remains relatively underexplored.

In this study, we developed and evaluated deep learning models for detecting corneal perforation in ASOCT images of eyes with microbial keratitis. We compared model configurations to assess whether inclusion of healthy control eyes in training datasets or masking of non-corneal anatomy affected diagnostic performance. This study adhered to the Standards for Reporting Diagnostic Accuracy Studies for Artificial Intelligence (STARD-AI) guidelines [25].

## 2. Materials and Methods

### 2.1. Study Design and Ethics

**Figure 1:**
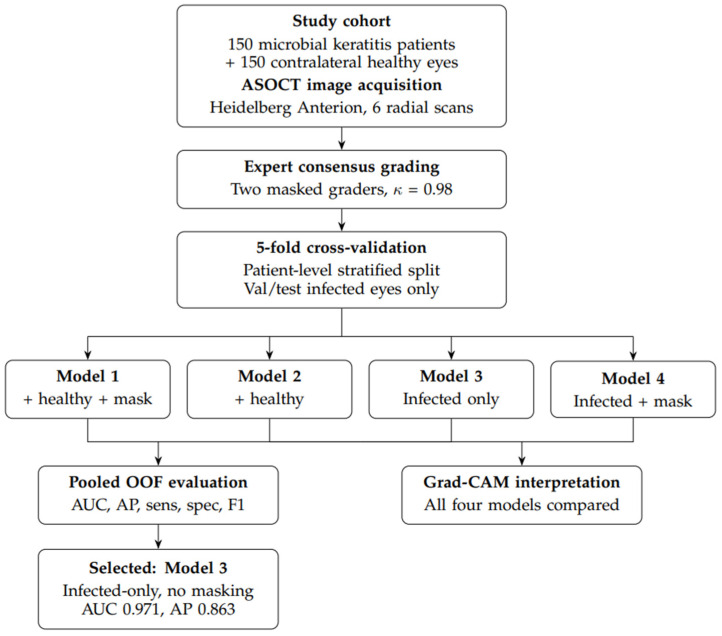
Study workflow. Schematic overview of patient enrollment, ASOCT image acquisition with the Heidelberg Anterion (six radial scans per eye), expert consensus grading by two masked ophthalmologist graders (κ = 0.98), patient-level stratified 5-fold cross-validation with validation and test splits restricted to infected eyes, training of four model variants differing in inclusion of contralateral healthy controls and stochastic inferior blackout masking, pooled out-of-fold (OOF) evaluation with Grad-CAM interpretability analysis, and selection of the best-performing configuration (Model 3).

This was a cross-sectional diagnostic accuracy study evaluating four deep learning models for the detection of corneal perforation on ASOCT imaging. The index test consisted of convolutional neural network-based classification models, and the reference standard was expert consensus grading of perforation status based on multimodal clinical imaging. This study adhered to the tenets of the Declaration of Helsinki and received approval from the Johns Hopkins University School of Medicine institutional review board and the SNC Chitrakoot Eye Hospital institutional research ethics committee. All participants provided written informed consent prior to study enrollment. An overview of the study workflow is provided in Figure 1.

### 2.2. Study Population

We enrolled consecutive patients with microbiologically confirmed bacterial or fungal keratitis treated at SNC Chitrakoot, a tertiary eye care center in Madhya Pradesh, India, between May 2024 and December 2024. Eligible patients had bacterial, fungal, or polymicrobial keratitis confirmed via microbiologic culture or smear microscopy testing. We excluded eyes with ungradable image quality, unreadable ASOCT files, or missing slit lamp examination findings. Contralateral eyes of enrolled patients without evidence of corneal infection, scarring, or perforation were used as healthy controls. Data from baseline visits were included in the algorithm training and evaluation datasets.

### 2.3. ASOCT Image Acquisition

ASOCT imaging was performed using the Heidelberg Anterion platform (Heidelberg Engineering, Heidelberg, Germany) with the Metrics App, which obtains six radial cross-sectional scans that capture limbus-to-limbus images of the cornea and anterior segment anatomy. This standardized imaging protocol provided comprehensive sampling across multiple evenly spaced meridians of the front of the eye. Additional clinical contextual information used during human grading of ASOCT images included diffuse illumination slit lamp photographs, cobalt blue illumination photographs taken 10–15 s after instillation of fluorescein dye, and grayscale photographs of the cornea taken using the Heidelberg Anterion device with a superimposed reference line indicating the exact anatomic cross-section captured on the ASOCT scan (Supplementary Figure S1).

### 2.4. Expert Ground-Truth Labeling of ASOCT Images

For each eye, two masked ophthalmologist graders independently evaluated all six radial ASOCT images alongside ancillary imaging and graded the eye for the presence or absence of frank corneal perforation. Frank corneal perforation was defined as a full-thickness defect of the stroma with Descemet membrane discontinuity; a collapsed or nearly collapsed anterior chamber with large cross-sectional areas of iris–cornea touch in all imaging meridians, along with evidence of severe corneal thinning and/or descemetocele; and/or overt visualization of iris tissue plugging an area of perforated cornea. Old perforations that had healed with corneal re-epithelialization were not classified as frank perforations. Eyes with iris synechiae extending to the posterior cornea but with a formed anterior chamber and no evidence of severe corneal thinning were also not classified as having active perforation. Following independent grading, disagreements between graders were resolved via a consensus discussion. Inter-grader agreement for ASOCT-based perforation detection was near-perfect (κ = 0.98; 95% CI, 0.92–1.00), with only 1 of 150 images (0.7%) requiring adjudication, as previously reported [21]. Active corneal perforation was defined as an eye-level diagnosis due to its effect on the entire anterior chamber anatomy and because of the possibility of the six radial cuts failing to capture the exact location of the full-thickness corneal defect.

### 2.5. Deep Learning Model Development

**Figure 2:**
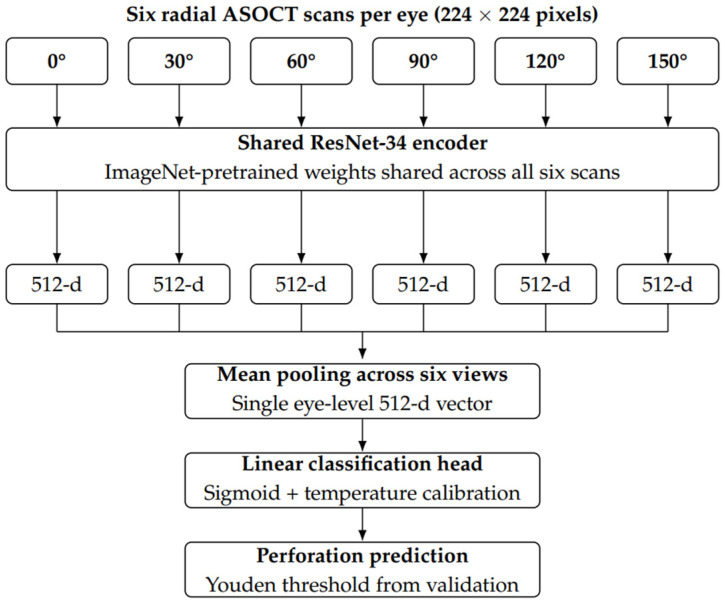
Deep learning model architecture. Each of the six radial ASOCT scans per eye (0°, 30°, 60°, 90°, 120°, 150°) is resized to 224 × 224 pixels and passed independently through a shared ResNet-34 [26] encoder pretrained on ImageNet [27], producing a 512-dimensional feature vector for each scan. The six per-scan vectors are mean-pooled into a single eye-level representation and mapped to a scalar perforation probability by a linear classification head, followed by post hoc temperature calibration [28] on the validation split. The operating threshold is selected by the validation-derived Youden index [29].

A deep convolutional neural network was trained to predict the presence of corneal perforation from ASOCT images. Each eye was represented by six radial ASOCT images, and all scans from the same eye were aggregated to produce a single eye-level prediction. A ResNet-34 [26] backbone pretrained on ImageNet [27] was used as a shared feature extractor. Each radial scan was treated as an individual single-channel image and passed independently through the shared ResNet-34 [26] encoder. To accommodate single-channel ASOCT images, the first convolutional layer was modified from a three-channel input to a one-channel input convolution, with pretrained weights preserved by averaging across the original RGB channels. The encoder’s global average-pooling layer reduced each scan to a 512-dimensional feature vector. The six scan-level feature vectors were mean-pooled to form a single eye-level representation and mapped to a scalar logit by a linear classification head. This design preserves the information from all six views for joint reasoning through the shared extraction and aggregation (Figure 2).

Four model variants were trained and evaluated. Throughout this manuscript, training cohorts are referred to as the “+healthy” cohort (training set augmented with contralateral healthy eyes) and the “infected” cohort (training set restricted to eyes with microbial keratitis). The test set was always restricted to eyes with microbial keratitis and is referred to as the “keratitis-only test cohort”:

1. **Model 1:** +healthy training cohort; inferior portion of image randomly masked during training to exclude lens anatomy.
2. **Model 2:** +healthy training cohort; no image masking applied.
3. **Model 3:** Infected training cohort; no image masking applied.
4. **Model 4:** Infected training cohort; inferior portion of image randomly masked during training to exclude lens anatomy.

For each eye, one scan was loaded from each angle (0°, 30°, 60°, 90°, 120°, and 150°). The inputs were resized to 224 × 224 pixels, and per-fold input normalization was computed on the training-split infected keratitis cohort scans only for each cross-validation fold, and the same statistics were applied at validation and test time. During training, data augmentation included random horizontal flipping with probability *p =* 0.5, random rotation of ±10°, and, for Models 1 and 4, stochastic blackout masking of the inferior image portion. For each training sample, the masking boundary was drawn uniformly from [70, 115] pixels and applied with a probability of 0.5. Pixels below the boundary were set to 0. No masking was applied at validation or test time. Models were trained for 50 epochs with a batch size of 16 using the AdamW optimizer with weight decay of 1 × 10^−3^. Learning rate scheduling was performed using ReduceLROnPlateau based on validation loss. Binary cross-entropy was used as the training objective, and weighted random sampling was used to handle class imbalance. Checkpoint selection for evaluation was done using the validation AP. After model selection, post hoc temperature scaling [28] was fit on the validation split, and the validation-derived Youden index [29] was computed to provide threshold-dependent metrics. Training was performed on a single NVIDIA RTX A5500 GPU. All reported 95% CIs are from patient-cluster bootstrap, in which the resampling unit is the patient (not the eye), with n = 2000 resamples for tabular metrics.

To ensure full reproducibility, all random seeds were set to 0. This included the seeds governing patient-level fold assignment, weighted random sampler initialization, model parameter initialization, and stochastic data augmentation operations (horizontal flip, rotation, and inferior blackout masking).

### 2.6. Data Partitioning

Because contralateral healthy eyes come from the same patients as keratitis eyes, data were partitioned at the patient level to prevent data leakage, ensuring that images from the same patient did not appear in both training and test sets. Patient-level stratification was used to preserve the distribution of perforation cases across folds. The dataset was divided into five folds. In each cross-validation iteration, one fold was held out as the test set, the next fold served as the validation set for checkpointing by validation AP, post hoc temperature calibration [28], and Youden threshold selection [29], and the remaining three folds were used for training. This process was repeated so that each fold served as the test set once. Model checkpoints within each iteration were selected based on the validation AP. Table 1 summarizes the patient-level split sizes and the number of perforation cases in each cross-validation iteration.

### 2.7. Statistical Analysis

Model performance was evaluated using ROC AUC, sensitivity, specificity, F_1_ score, and AP. Ninety-five percent confidence intervals were calculated using bootstrap resampling. Calibration was assessed using calibration curves comparing predicted probabilities to observed outcome frequencies and the Brier score and expected calibration error (ECE). ECE was computed using 5 equal-width bins; we used 5 bins rather than the more common 10 because of the limited test set size (n = 30 patients per fold). With 10 bins, the small number of positive cases per fold (4–5 perforation events) would yield sparsely populated or empty bins, producing unstable and high-variance estimates. Five bins preserve the interpretability of the calibration estimate while maintaining adequate sample density in each bin. Optimal classification thresholds were determined using the Youden index [29] in the validation split. Gradient-weighted class activation mapping (Grad-CAM) [30] was used to generate heatmaps visualizing regions of input images that contributed most to model predictions.

## 3. Results

### 3.1. Study Population

Table 2 shows demographic and clinical characteristics for the study population, with additional clinical details provided in Supplementary Table S1. A total of 150 eyes from 150 patients with microbiologically confirmed microbial keratitis were included in the analysis. The median age was 50.0 years (IQR, 41.0–59.0), and 92 patients (61.3%) were male. Right eyes comprised 64.0% of the study population. The majority of patients (80.0%) presented with visual acuity of logMAR 1.0 or worse, indicating the severity of the presenting disease in this high-risk, low-resource rural population. The median duration from symptom onset to presentation was 15.0 days (IQR, 7.0–30.0 days).

Fungal keratitis was the most common infection type (80 eyes, 53.3%), followed by bacterial keratitis (54 eyes, 36.0%) and polymicrobial infections (16 eyes, 10.7%). Most infiltrates measured 2–6 mm in diameter (69.3%), with 26.0% demonstrating posterior one-third stromal involvement. Forty-six eyes (30.7%) had stromal thinning severe enough to be evident on slit lamp examination. Based on consensus ASOCT grading, 24 eyes (16.0%) had frank corneal perforation, and 126 eyes (84.0%) did not have perforation. Contralateral healthy control eyes without active corneal infection (N = 150) were included in training datasets for Models 1 and 2.

### 3.2. Model Performance

**Figure 3:**
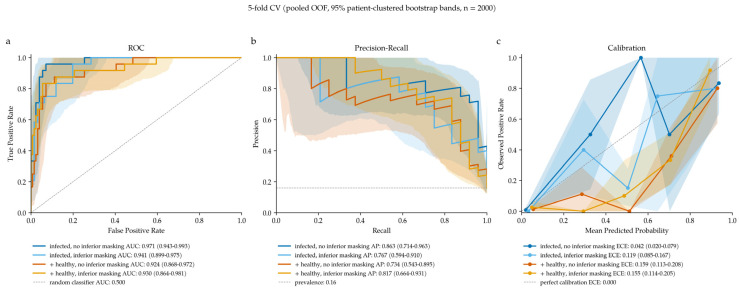
Discrimination and calibration plots for the four deep learning models. (**a**) Receiver operating characteristic (ROC) curves. (**b**) Precision–recall curves. (**c**) Calibration curves. Shaded bands denote 95% patient-clustered bootstrap intervals (n = 2000) computed on pooled out-of-fold (OOF) test predictions across the 5-fold cross-validation.

**Figure 4:**
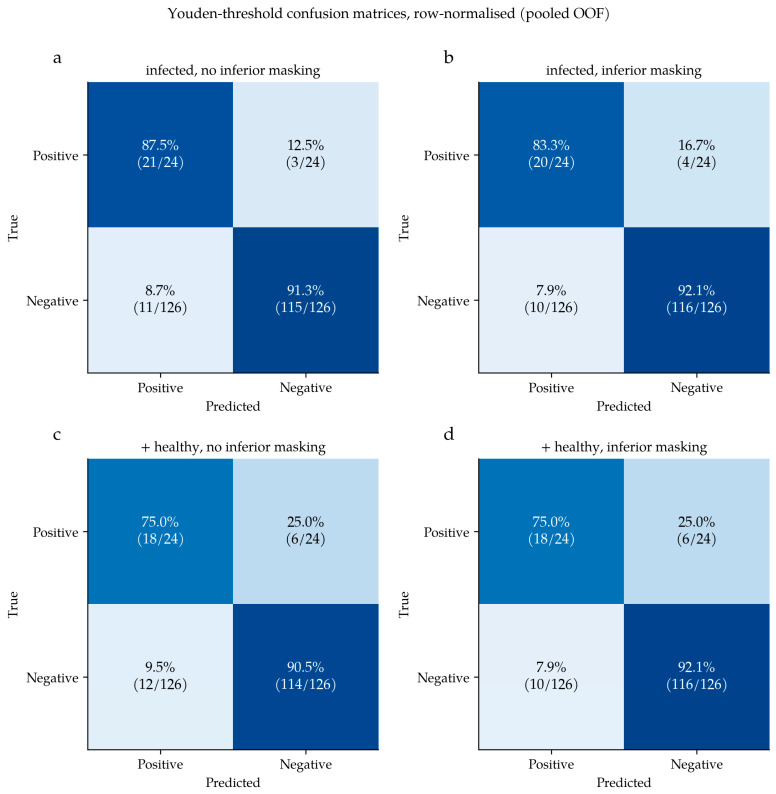
Row-normalized confusion matrices for the four deep learning models at validation-derived Youden thresholds [29], computed on pooled OOF predictions. (**a**) Model 3 (infected cohort, no inferior masking). (**b**) Model 4 (infected cohort, inferior masking). (**c**) Model 2 (+healthy cohort, no inferior masking). (**d**) Model 1 (+healthy cohort, inferior masking).

Model performances are summarized and compared in Table 3, with receiver operating characteristic curves, precision–recall curves, calibration curves, and confusion matrices for each model shown in Figure 3 and Figure 4. Model 1 (+healthy cohort, masking of deeper anatomy) achieved an ROC AUC of 0.930 (95% CI, 0.864–0.981) and AP of 0.817 (95% CI, 0.664–0.931). The sensitivity was 75.0% (95% CI, 55.0–90.9%), and the specificity was 92.1% (95% CI, 87.3–96.7%). The F1 score was 69.2% (95% CI, 52.2–82.4%). Model 2 (+healthy cohort, no image masking) achieved an ROC AUC of 0.924 (95% CI, 0.868–0.972), sensitivity of 75.0% (95% CI, 55.6–91.3%), and specificity of 90.5% (95% CI, 85.1–95.3%). The F1 score was 66.7% (95% CI, 50.0–80.0%), and the average precision was 0.734 (95% CI, 0.543–0.895). Model 3 (infected cohort, no image masking) achieved an ROC AUC of 0.971 (95% CI, 0.943–0.993), sensitivity of 87.5% (95% CI, 72.7–100.0%), specificity of 91.3% (95% CI, 85.8–95.9%), F1 score of 75.0% (95% CI, 60.9–87.0%), and AP of 0.863 (95% CI, 0.714–0.963). Model 4 (infected cohort, masking of deeper anatomy) achieved an ROC AUC of 0.941 (95% CI, 0.899–0.975), sensitivity of 83.3% (95% CI, 66.7–96.0%), specificity of 92.1% (95% CI, 86.9–96.7%), F1 score of 74.1% (95% CI, 58.8–86.2%), and average precision of 0.767 (95% CI, 0.594–0.910). Across all four model variants, the best overall discriminative performance was observed when healthy controls were excluded, and no image masking was applied during training (Model 3), which achieved the highest ROC AUC, AP, sensitivity, and F1 score, though the 95% CI for ROC AUC overlapped across all four models.

### 3.3. Grad-CAM Visualization

**Figure 5:**
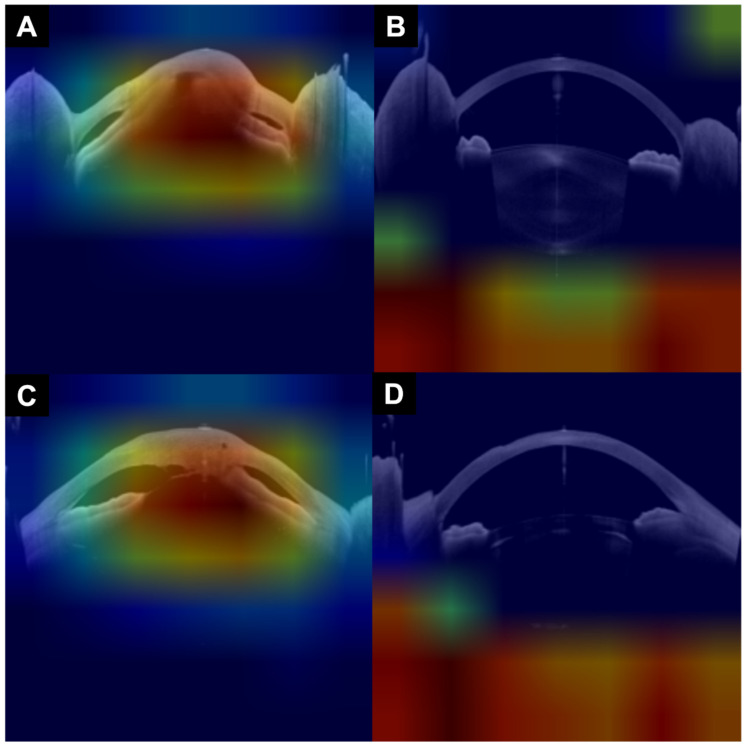
Representative Grad-CAM heatmaps demonstrating patterns of model attention across classification outcomes. Warmer colors indicate greater attention. Cooler colors indicate less attention. Figure 5 legend: (**A**) Model 1 (+healthy cohort, inferior masking) true positive: attention focused on anterior chamber collapse and iris–cornea touch in an eye with frank perforation, demonstrating that the model learned clinically relevant anatomic features for perforation detection. (**B**) Model 2 (+healthy cohort, no masking) true negative: attention focused on the lens and structures posterior to the lens in a non-perforated eye, reflecting learned association between visibility of deeper anatomy and absence of perforation. This pattern emerged because non-visible lens anatomy occurs more frequently in eyes with severe disease and media opacity, while healthy control eyes have readily visible lens anatomy. (**C**) Model 3 (infected cohort, no masking) false positive: attention on iris–cornea touch from anterior synechiae in a non-perforated eye, illustrating the principal failure mode in which the model misclassified eyes with shallow anterior chambers or synechiae that falsely mimic the anatomy of eyes with corneal perforation. (**D**) Model 4 (infected cohort inferior masking) true negative: attention is focused on the inferior region of the image located at or posterior to the lens, despite this region being masked during training. Also observed in Model 1, this pattern’s mechanism remains unclear and warrants further investigation. Of note, Models 3 and 4 exhibited spatially diffuse activation patterns overall, reflecting the absence of healthy control comparators during training, which may have prevented the model from learning to distinguish cornea-specific features from non-specific image characteristics.

Grad-CAM heatmaps demonstrated distinct patterns of attention across the four models (Figure 5; Supplementary Table S2). In true positive predictions among eyes with perforation, all four models consistently attended to the anterior chamber, anteriorly displaced iris tissue, and/or areas of iris–cornea touch from collapsed iridocorneal angles, anterior synechiae, or iris plugging of corneal stromal defects. False positives across all models were characterized by attention to iris–cornea touch in eyes with anterior synechiae or shallow anterior chambers without frank perforation. True positives in all four models tended to show minimal activation over deeper non-corneal structures such as the lens. False negatives, though rare, occurred when models attended to structures at or posterior to the lens rather than the anterior chamber. True negative predictions showed the most notable differences in heatmaps across model configurations. Models 1 and 2, which were trained with the +healthy cohort, demonstrated consistent attention to the region at or posterior to the lens. Models 3 and 4, trained with the infected cohort, showed more diffuse attention patterns with low spatial specificity, though Model 4 demonstrated somewhat more spatially consistent attention than Model 3.

Adherence to the STARD-AI reporting guidelines [25] is summarized in Supplementary Table S3.

## 4. Discussion

This study demonstrates that deep learning models can achieve high diagnostic accuracy for automated detection of corneal perforation on ASOCT imaging of eyes with microbial keratitis. The best-performing model achieved a pooled OOF ROC AUC of 0.971 (95% CI 0.943–0.993), establishing proof-of-concept that global eye-level assessments of perforation status can be reliably made using convolutional neural network architectures applied to ASOCT images.

### 4.1. Impact of Model Configuration on Performance

Our systematic comparison of four model configurations yielded insights relevant to the design of artificial intelligence systems for the assessment of anterior segment imaging. First, the inclusion of contralateral healthy control eyes in the training set did not improve any pooled OOF test metric. ROC AUC, AP, F1, Brier, and ECE were all best in the keratitis-only training cohort. A plausible reason could be the distribution shift between training and evaluation. With the inclusion of healthy contralateral eyes in training, the negative class becomes bimodal (healthy eyes and infected eyes without perforation), but the validation and test splits are restricted to the infected cohort only to allow for fair performance comparison across model variants. This finding has practical implications for curation of training datasets in future studies and may suggest that healthy controls are not ideal negative examples when the intended clinical task is distinguishing perforated from non-perforated infected eyes. Training datasets for this use case may instead benefit from prioritizing clinically realistic negative examples, including severe non-perforating ulcers, shallow anterior chambers, anterior synechiae, dense infiltrates, and other findings that may mimic perforation.

Second, stochastic masking of the inferior image region during training to exclude the inferior portion of images containing deeper anterior segment anatomy, such as the lens, produced opposing effects in the two training cohort settings. ASOCT images can contain complex anatomic information of regions beyond the cornea, including structural details about the anterior chamber, iris, iridocorneal angle, lens, and, in some cases, even the anterior vitreous. The lens region alone exhibits substantial variability across eyes due to the presence of cataract, pseudophakia, aphakia, or imaging artifacts, none of which are relevant to detecting corneal perforation. By training models to ignore the lens region, we reduced the potential for spurious correlations. However, the stochastic masking decreased ROC AUC and AP in the infected cohort. In the +healthy cohort, stochastic masking helped both ROC AUC and AP. A plausible interpretation is that the stochastic masking during training acts as a regularizer and may help when the training set distribution is broader by preventing overfitting to lens-region features that vary across eyes. Conversely, masking may be less useful or potentially detrimental when the training set distribution is already closely matched to the evaluation cohort, because it may remove contextual information useful for distinguishing perforated from non-perforated infected eyes.

Although Model 3 achieved the highest point estimates across nearly all metrics, 95% confidence intervals for ROC AUC and AP overlapped substantially across all four model variants. Recommendations regarding the optimal configuration should therefore be interpreted as suggestive and viewed as hypothesis-generating and useful for guiding future model development, dataset curation, and validation experiments rather than immediate selection of a single optimal configuration.

Grad-CAM visualization results provide insight into the differential performance across models (Figure 5; Supplementary Table S2). All four models attended to clinically meaningful anterior segment structures in true positive predictions, including anterior chamber collapse, anteriorly displaced iris tissue, and iris–cornea touch. This consistency suggests that the models learned anatomic features relevant to detecting perforation regardless of model training configuration. The principal failure mode across all models was false positive classification of eyes with iris–cornea touch (anterior synechiae) or shallow anterior chambers mimicking perforation anatomy, though this occurred infrequently, given model specificities ranging from 90.5% to 92.1%.

The most notable differences across model configurations were evident in true negative predictions. For Model 2, which did not employ inferior masking, consistent attention to the region at or posterior to the lens likely reflects learned attention to lens visibility as a discriminative feature, since healthy corneas enable visualization of deeper anatomy, whereas opaque or perforated corneas do not. However, lens obscuration can also occur with severe non-perforating conditions such as dense infiltrates or corneal edema, making this an indirect marker that could limit generalizability. Models 3 and 4, trained without healthy controls, showed more diffuse attention in true negatives, consistent with weaker baseline representations of normal anatomy.

Interestingly, both models with inferior masking (Models 1 and 4) demonstrated attention to the region at or posterior to the lens in true negatives, despite this region being masked during training. The mechanism underlying this pattern is unclear. The models may have learned to recognize the masked region itself as an informative feature, or the masking may have influenced how information from adjacent unmasked regions is encoded. Further research using alternative interpretability methods or controlled ablation experiments is needed to clarify how inferior masking affects learned feature representations and whether attention to deeper anterior segment structures reflects clinically meaningful information or shortcut learning.

### 4.2. Clinical Implications

Corneal perforation in the setting of microbial keratitis represents a true ophthalmic emergency, often requiring urgent procedural intervention [5,14]. Despite its seriousness, detection of corneal perforation can be challenging when severe corneal infiltrates, scarring, or edema obscure direct visualization of stromal integrity on slit lamp examination. These diagnostic challenges are worse for non-cornea specialists who may lack experience in the assessment of corneal infections. Prior work has demonstrated that human graders evaluating ASOCT images can detect substantially more perforations than clinical examination, with slit lamp examination demonstrating only 33.3% sensitivity when ASOCT is used as the reference standard [15]. Ophthalmologist graders also show high intra- and inter-grader repeatability for perforation detection on ASOCT images, indicating that such assessments can yield objective and reproducible labels for model training. Assuming ophthalmologist assessments can be replicated by computer vision, an automated system for perforation detection could serve as a scalable clinical decision-support tool to identify high-risk cases needing urgent specialist review, particularly in settings lacking specialist expertise in corneal disease or ASOCT interpretation. As ASOCT technology becomes more portable, affordable, and able to visualize larger cross-sections of the anterior segment, ASOCT systems equipped with automated diagnostic algorithms could conceivably be deployed to enable infection and trauma diagnosis at the frontlines of eye care, such as emergency departments, urgent care clinics, and community eye screening programs. Given that expert human grading of ASOCT enables superior detection of perforation than slit lamp examination [15], ASOCT-based automated perforation detection could even improve the quality of care in tertiary care centers relative to specialist-performed slit lamp examination and could also enable consistently improved diagnosis relative to specialists who may show varying diagnostic performance when interpreting ASOCT images.

### 4.3. Implications for Future Models for ASOCT Interpretation

Several implications emerge for the development of future AI systems for anterior segment OCT analysis. First, our findings suggest that uncritical inclusion of negative-control eyes (healthy contralateral eyes) in training does not necessarily improve performance when the deployment population is restricted to diseased eyes. Training cohort composition should therefore match the intended evaluation distribution. Second, deliberate anatomic masking may help focus model attention on clinically relevant regions and reduce model overfitting to spurious correlations. Third, the use of Grad-CAM and similar explainability methods can provide valuable insights into model decision-making that inform iterative model refinement. Future work could explore attention mechanisms or region-of-interest architectures that more explicitly encode anatomical priors. This approach could be extended to other anterior segment structures and conditions detectable on ASOCT images, such as hypopyon, endothelial plaque, descemetocele formation, and iridocorneal lesions. Integration of multimodal imaging data, such as slit lamp photographs and/or Scheimpflug tomography, may further improve model performance.

### 4.4. Strengths and Limitations

This study has several strengths. The ground-truth labels were established through rigorous masked grading by ophthalmologists who achieved near-perfect inter-grader agreement (κ = 0.98), meaning the model was trained using a highly reliable reference standard. Our systematic comparison of four different model configurations enabled mechanistic insights into factors influencing model performance. The use of Grad-CAM visualization produced interpretable evidence supporting our hypotheses about model behavior.

Algorithmic fairness is a key consideration for AI systems intended for clinical deployment [25]. Our model was trained on data from a rural population in central India, the country with the highest absolute burden of microbial keratitis worldwide [31]. Patients in our dataset presented with clinical characteristics typical of resource-limited settings, including delayed presentation (median 15 days from symptom onset) and a high proportion of fungal infection. This training approach ensures that model development is aligned with the population where AI-assisted perforation detection has substantial potential clinical utility. The anatomic features used by the model to detect perforation, such as full-thickness stromal defects and anterior chamber architecture, are structural findings on ASOCT imaging that are not expected to vary systematically by demographic or geographic factors. Although external validation across heterogeneous clinical settings is required before deployment, training on data from a high-burden population may enhance generalizability to similar settings, where the need for automated diagnostic support is high.

Several limitations warrant consideration. First, this study was conducted at a single tertiary referral center in India that serves a rural, low-resource population presenting with severe stages of disease. Although the higher baseline prevalence of perforation in the resulting dataset was ideal for training deep learning models for perforation detection, algorithm performance should be externally validated across heterogeneous geographic, demographic, and clinical settings to verify generalizability prior to clinical deployment. Future studies should train and evaluate the performance of ASOCT-based perforation detection algorithms in larger, multicenter datasets. Second, our algorithms were only trained to detect frank perforation, meaning that the algorithms were not trained to detect micro-perforations, previously healed perforations, or various forms of profound anatomic damage without perforation, such as severe thinning, descemetoceles, iridocorneal synechiae, or posterior staphylomas. Future research should evaluate algorithm performance for identifying these other clinical features, which can also inform clinical and surgical management. Third, because the Heidelberg Anterion ASOCT hardware and imaging settings use specific scan parameters, our results may not be generalizable to other ASOCT devices with different imaging characteristics. Fourth, the relatively small number of perforation cases (n = 24) limited statistical power for subgroup analyses and may have affected model calibration. Fifth, we evaluated models using cross-validation rather than a held-out prospective test set and selected an operating threshold via the Youden index [29] on the validation fold, which may overestimate performance compared to real-world deployment. Sixth, the Grad-CAM analysis in this study was qualitative. Quantitative metrics like Dice and IoU were not computed and would require substantial work for expert-level annotation and agreement. Establishing reproducible spatial ground truth and computing localization metrics against such ground-truth labeling is an important direction for future work. Seventh, this study assessed model accuracy without evaluating the impact of AI assistance on clinical decision-making in real-world settings. In order for an ASOCT perforation algorithm to be considered appropriate for clinical use, this study should be replicated for a larger dataset with a hold-out test set and a greater number of perforation-positive cases per fold in order to achieve narrower confidence intervals. Algorithm performance should also be externally validated to assess generalizability across heterogeneous demographic and geographic populations, across low- and high-resource healthcare settings, and by subgroups such as infection type, severe versus non-severe infections, early- versus late-stage disease, and across different ASOCT machines. Future studies should also evaluate algorithm performance against perforation diagnoses made by clinicians with varying levels of expertise, grading prospectively collected images. Nonetheless, our analyses should be considered proof-of-concept that an ASOCT-based perforation detection algorithm has feasibility and potential for clinical utility in the future.

## 5. Conclusions

Deep learning models achieved high diagnostic accuracy for detecting corneal perforation on ASOCT imaging in patients with microbial keratitis, with the recommended model achieving an ROC AUC of 0.971. Masking of non-corneal anatomy had opposing effects on model performance in the two different training cohort settings. Our findings establish the feasibility of automated ASOCT analysis as a potential clinical decision-support tool for identifying this vision-threatening complication of microbial keratitis.

## Figures and Tables

**Table 1:** Cross-validation splits.

Iteration	Train Set (Total Patients/Positive Perforation)	Validation Set (Total Patients/Positive Perforation)	Test Set (Total Patients/Positive Perforation)
**0**	90/15	30/5	30/4
**1**	90/14	30/5	30/5
**2**	90/14	30/5	30/5
**3**	90/14	30/5	30/5
**4**	90/15	30/4	30/5

**Table 2:** Demographic and clinical characteristics of the study population.

Characteristics	Value (N = 150)
**Age, median (IQR)**	50.0 (41.0–59.0)
**Sex**	
Female, N (%)	58 (38.7)
Male, N (%)	92 (61.3)
**Eye Laterality**	
Right eye, N (%)	96 (64.0)
Left eye, N (%)	54 (36.0)
**LogMAR VA, median (IQR)**	1.39 (0.6–2.0)
**Perforation Status on ASOCT**	
Present, N (%)	24 (16.0)
Absent, N (%)	126 (84.0)
**Infection Type**	
Fungal Only	80 (53.3)
Bacterial Only	54 (36.0)
Polymicrobial	16 (10.7)
**Stromal Thinning, N (%)**	46 (30.7)
**Days to Presentation, median (IQR)**	15.0 (7.0–30.0)

Abbreviations: ASOCT = anterior segment optical coherence tomography; IQR = interquartile range; logMAR = logarithm of minimum angle of resolution; N = number; VA = visual acuity.

**Table 3:** Comparison of deep learning model performance. Metrics are reported from the OOF mean test (95% CI).

	Model 1 (+Healthy, Inferior Masking)	Model 2 (+Healthy, No Masking)	Model 3 (Infected, No Masking)	Model 4 (Infected, Inferior Masking)
**ROC AUC**	0.930 (0.864–0.981)	0.924 (0.868–0.972)	0.971 (0.943–0.993)	0.941 (0.899–0.975)
**AP**	0.817 (0.664–0.931)	0.734 (0.543–0.895)	0.863 (0.714–0.963)	0.767 (0.594–0.910)
**Sensitivity at 0.5**	0.875 (0.722–1.000)	0.875 (0.722–1.000)	0.792 (0.611–0.941)	0.833 (0.667–0.962)
**Specificity at 0.5**	0.794 (0.722–0.864)	0.802 (0.734–0.870)	0.960 (0.926–0.992)	0.849 (0.787–0.907)
**F1 at 0.5**	0.592 (0.444–0.716)	0.600 (0.451–0.727)	0.792 (0.640–0.898)	0.635 (0.480–0.762)
**Sensitivity at Youden** [29]	0.750 (0.550–0.909)	0.750 (0.556–0.913)	0.875 (0.727–1.000)	0.833 (0.667–0.960)
**Specificity at Youden** [29]	0.921 (0.873–0.967)	0.905 (0.851–0.953)	0.913 (0.858–0.959)	0.921 (0.869–0.967)
**F1 at Youden** [29]	0.692 (0.522–0.824)	0.667 (0.500–0.800)	0.750 (0.609–0.870)	0.741 (0.588–0.862)
**Brier Temperature Scaled** [28]	0.114 (0.086–0.144)	0.119 (0.088–0.152)	0.050 (0.025–0.079)	0.091 (0.067–0.116)
**ECE Temperature Scaled** [28]	0.155 (0.114–0.205)	0.159 (0.113–0.208)	0.042 (0.020–0.079)	0.119 (0.085–0.167)

Abbreviations: AP = average precision, ROC AUC = area under the receiver operating characteristic curve.

## Data Availability

Datasets and code used in this study may be made available upon reasonable request and in accordance with local regulations. The trained model weights are available at https://github.com/lhrhode/ASOCT_Corneal_Perforation_Detection (accessed on 17 April 2026).

## Supplementary Materials

The following supporting information can be downloaded at https://www.mdpi.com/article/10.3390/bioengineering13060649/s1, Supplementary Table S1. Detailed clinical characteristics of the study population. Supplementary Table S2. Qualitative analysis of Grad-CAM heatmap patterns across model configurations and classification outcomes. Supplementary Table S3. STARD-AI checklist with manuscript location references. Supplementary Figure S1. Example of ASOCT composite images used for human labeling of perforation in microbial keratitis.
