# Supplementary material for: Deep Learning for the Detection of Corneal Perforation on Anterior-Segment Optical Coherence Tomography in Microbial Keratitis
Source: Bioengineering (Basel). 2026 May 30;13(6):649. doi: 10.3390/bioengineering13060649 (PMC13295327; doi:10.3390/bioengineering13060649)
Supplement: Supplementary file 1 [file bioengineering-13-00649-s001.zip › bioengineering-4296150-supplementary.pdf]

Table S1. Detailed Clinical Characteristics of Study Population.

Characteristics	Value (Total N=150)
**Snellen visual acuity, N (%)**	
6/6 to ≤6/18	17 (11.3)
6/24 to ≤6/36	13 (8.7)
1/60 to ≤6/60	32 (21.3)
Finger counts or Hand movements	36 (24.0)
Light perception or No light perception	52 (34.7)
**Lens Status, N (%)**	
Clear crystalline lens	57 (38.0)
Early lens changes	31 (20.7)
Immature cataract	31 (20.7)
Pseudophakia	8 (5.3)
Unable to determine	22 (14.7)
**Infiltrate Diameter**	
0 to <2mm	21 (14.0)
2 to <6mm	104 (69.3)
≥6mm	24 (16.0)
**Infiltrate Depth**	
Anterior 1/3 Stroma	133 (88.7)
Middle 1/3 Stroma	97 (64.7)
Posterior 1/3 Stroma	39 (26.0)
**Infiltrate Within 2mm of Limbus**	13 (8.7)

Abbreviations: mm = millimeter; N = number.

Table S2. Qualitative Analysis of Grad-CAM Heatmap Patterns Across Model Configurations and Classification Outcomes.

Model	Outcome	Primary Anatomic Region of Attention	Characteristic Attention Pattern	Clinical Interpretation
Model 1 (healthy controls, inferior masking)	True positive	Anterior chamber	Anterior chamber highlighted in nearly all true positive eyes; lens anteriorly displaced into position where anterior chamber would normally be located; broad iris-cornea touch regions activated	Model correctly identified anterior chamber collapse and iris displacement as markers of perforation
	True negative	At or posterior to lens	Nearly all eyes show attention over a horizontal region at or posterior to lens which is consistent with inferior masked region; occasional attention to abnormal iris anatomy in infected eyes	Model's consistent attention to inferior masked regions may reflect recognition of mask itself as an informative feature, or encoding of information from adjacent unmasked regions
	False positive	Anterior chamber	All false positives show attention over areas of iris-cornea touch and/or shallow anterior chambers without frank perforation	False positives occurred in eyes with anterior synechiae mimicking perforation anatomy

	False negative	Posterior to cornea	Attention focused on horizontal region posterior to cornea, more so than anterior chamber	Missed perforations occurred when model attended to deeper structures located at or posterior to the lens rather than within the anterior chamber
Model 2 (healthy controls, no masking)	True positive	Anterior chamber and lens region	Anterior chamber highlighted in all true positive eyes; anteriorly displaced lens and iris-cornea touch areas also activated	Similar detection pattern to Model 1
	True negative	At or posterior to lens	Nearly all eyes show attention over a horizontal region at or posterior to lens; some attention to iris-cornea touch or anteriorly bowed iris	Model learned that visibility of the lens and structures posterior to it indicates a non-perforated eye; healthy controls establish baseline representation of normal anatomy
	False positive	Iris-cornea interface	Attention focused on iris-cornea touch, with iris abnormalities and attention typically more prominent on one side of anterior chamber	Similar error mode to Model 1; anterior synechiae without frank perforation triggered false positive classification
	False negative	Lens region	Attention over region posterior to anteriorly displaced iris, in presumed location of lens	Model attended to lens region rather than recognizing anterior chamber collapse
Model 3 (no healthy controls, no masking)	True positive	Anterior chamber and iris-cornea touch	Attention focused on anterior chamber region posterior to anteriorly bowed iris, or directly over areas of iris-cornea touch	Correct anatomic attention, but model lacked baseline representation of normal anatomy
	True negative	Variable and diffuse	Less consistent pattern; many eyes show attention over region posterior to lens; some show attention over anterior chamber or areas of iris abnormality	Without healthy controls, model developed weaker representation of normal anatomy; attention patterns less spatially organized
	False positive	Posterior to iris	Attention over area posterior to anteriorly bowed iris	Similar error pattern to other models
	False negative	Iris abnormalities	Iris-cornea touch and iris abnormality highlighted	Model attended to relevant structures but failed to classify correctly
Model 4 (no healthy controls, inferior masking)	True positive	Anterior chamber	All true positive eyes show attention over anterior chamber; some eyes also show attention to areas of focal or broad iris-cornea touch occurring due to collapsed anterior chamber	Correct anatomic attention; inferior masking may have focused attention on anterior structures
	True negative	Posterior portion of image	Most eyes show attention over region posterior to lens; several eyes show attention over areas of focal iris abnormality such as synechiae or anteriorly bowed iris	More consistent attention than Model 3, suggesting inferior masking provided some regularization; however, attention to masked region despite its absence during training requires further investigation

False positive	Variable	Variable attention over iris-cornea touch, lens region posterior to bowed iris, or anterior chamber	Heterogeneous error patterns without single failure mode of mis-identification
False negative	Anterior chamber	Anterior chamber with focal iris-cornea touch highlighted	Model attended to correct anatomic region but still misclassified

Table S3. STARD-AI Checklist with Manuscript Location References.

	**TITLE/ABSTRACT**	
1	Identify as AI diagnostic accuracy study	Title, Abstract
2	Structured summary	Abstract
	**INTRODUCTION**	
3	Scientific/clinical background, intended use	Introduction
4	Study objectives and hypotheses	Introduction, para 4
	**METHODS**	
5	Study design	Methods: Study Design
6	Ethics approval and consent	Methods: Ethics
7	Eligibility criteria	Methods: Study Population
11	Data source and collection	Methods: ASOCT Acquisition
12	Dataset annotation	Methods: Reference Standard
13	Devices/software for index test	Methods: Model Development
14	Data acquisition protocols	Methods: ASOCT Acquisition
15b	Dataset partitioning	Methods: Data Partitioning
19	Reference standard definition	Methods: Reference Standard
20	Rationale for reference standard	Methods: Reference Standard
21-22	Statistical methods	Methods: Statistical Analysis
23	Algorithmic bias methods	Patient-level partitioning to prevent leakage and model evaluation across cross-validation folds with stratification for perforation status.
	**RESULTS**	
24	Flow of participants	Results: Study Population
25	Test set characteristics	Results: Study Population
26-27	Cross-tabulation and accuracy	Results: Model Performance, Table 3
28	Test set representativeness	Results: Study Population
29	Performance error analysis	Results: Grad-CAM
	**DISCUSSION**	
33-34	Limitations, implications	Discussion: Strengths/Limitations
35	Fairness considerations	Discussion: Strengths/Limitations
	**OTHER**	
36	Study registration	N/A
37-38	Protocol, funding	Funding section
39	Commercial interests	Disclosures
40a-b	Data/code availability, audit	Data Availability

This checklist follows the STARD-AI reporting guideline (Sounderajah V, et al. Nat Med. 2025;31:3283-3289).

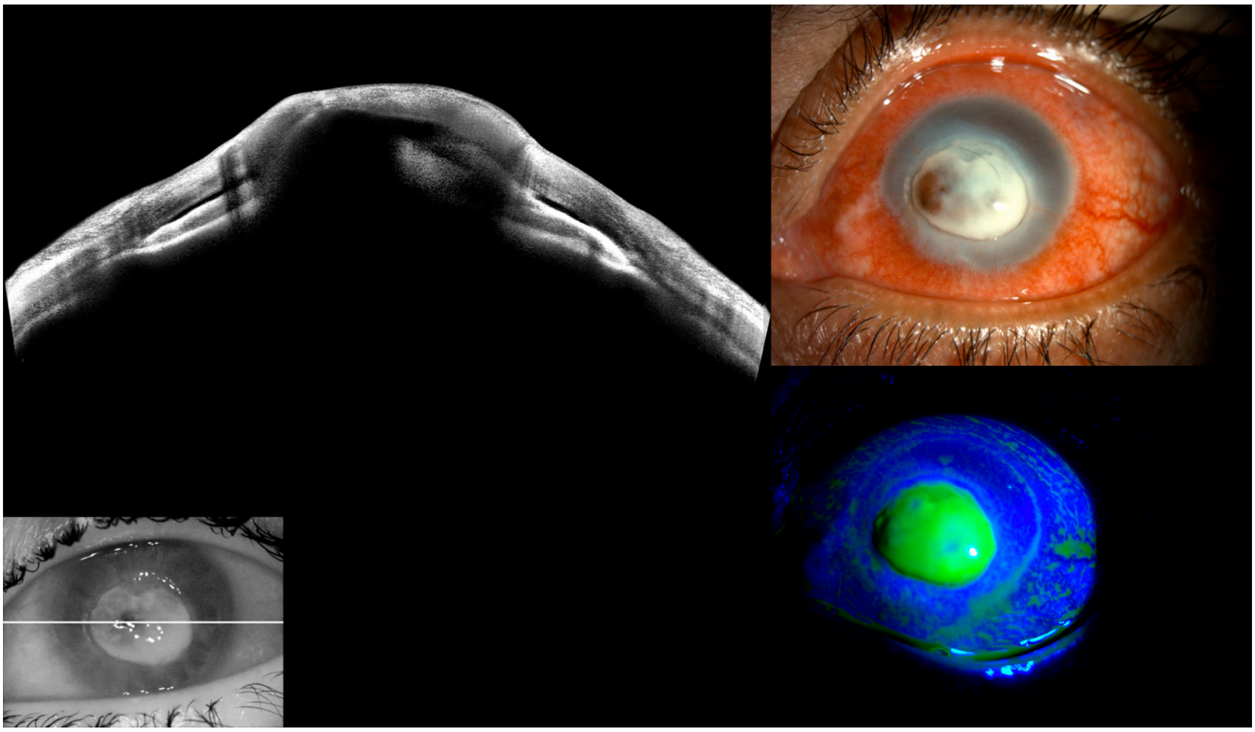

Figure S1. Example Of ASOCT Composite Images Used For Human Labeling Of Perforation In Microbial Keratitis. Supplementary Figure 1 legend: Composite images included an ASOCT radial scan (top-left), diffuse illumination slit lamp camera photograph of the eye (top-right), a cobalt blue light slit lamp camera photograph of the eye (bottom-right), and a black-and-white reference thumbnail showing location of radial cut (bottom-left). Each eye had six radial ASOCT scans, each with its own composite image. All six scans were assessed by two expert ophthalmologist graders to achieve an eye-level label of perforation versus no perforation. Disagreement between graders was resolved by repeat image review and discussion to achieve consensus. Final consensus labels were used for model training.
